# Non-Inversion Variants in Sporadic Hemophilia A Rarely Recur

**DOI:** 10.3390/ijms27093831

**Published:** 2026-04-25

**Authors:** Shih-Yao Lin, Ming Chen, Shun-Ping Chang, Gwo-Chin Ma, Dong-Jay Lee, Adeline Yan, Han-Ni Hsieh, Ming-Ching Shen

**Affiliations:** 1 AltruBio Inc., Taipei 115, Taiwan; shihyao.lin@altrubio.com; 2 Department of Genomic Medicine, Changhua Christian Hospital, Changhua 500, Taiwan; 104060@cch.org.tw (M.C.); 70914@cch.org.tw (S.-P.C.); 128729@cch.org.tw (G.-C.M.); 118862@cch.org.tw (D.-J.L.); 181435@cch.org.tw (A.Y.); 3 Department of Medical Genetics, National Taiwan University Hospital, Taipei 100, Taiwan; 4 Department of Obstetrics and Gynecology, Changhua Christian Hospital, Changhua 500, Taiwan; 5 Department of Obstetrics and Gynecology, National Taiwan University Hospital, Taipei 100, Taiwan; 6 Hemophilia Treatment and Thrombosis Center, Department of Internal Medicine, Changhua Christian Hospital, Changhua 500, Taiwan; 140660@cch.org.tw; 7 Department of Laboratory Medicine, National Taiwan University Hospital, Taipei 100, Taiwan; 8 Department of Internal Medicine, National Taiwan University Hospital, Taipei 100, Taiwan

**Keywords:** sporadic hemophilia A, non-inversion variant (NIV), linkage analysis, ARMS-qPCR

## Abstract

Hemophilia A (HA), an X-linked bleeding disorder caused by Factor VIII (*F8*) gene mutations, persists in the population due to sporadic cases arising from de novo mutations. This study analyzed 24 pedigrees from hemophilia registries of multiple medical centers in Taiwan to assess whether sporadic non-inversion variants (NIVs) recur in the same generation within families. Linkage analysis using intragenic and extragenic markers combined with amplification refractory mutation system-quantitative polymerase chain reaction (ARMS-qPCR) revealed that among 16 individuals inheriting the same X chromosome as the one bearing the sporadic HA-causing mutation, none carried the *F8* variant. These findings strongly suggest that sporadic NIVs exhibit very low risk of recurrence. Our results provide critical information for recurrence risk assessment and counseling strategies for HA sporadic NIVs.

## 1. Introduction

Factor VIII (FVIII), a cofactor comprising 2332 amino acids, facilitates the activation of Factor X (FX) by activated Factor IX (aFIX) during the coagulation cascade [1,2,3]. FVIII is encoded by the *F8* gene located on chromosome Xq28 [4]. Deficiency or dysfunction of FVIII due to *F8* gene mutations underlies hemophilia A (HA), the most common X-linked congenital bleeding disorder [5]. Clinical severity correlates with plasma FVIII activity: patients with mild (FVIII activity > 5–40 IU/dL) and moderate (FVIII activity 1–5 IU/dL) hemophilia may remain asymptomatic or have prolonged bleeding after minor trauma whereas those with severe hemophilia (FVIII activity < 1 IU/dL) often experience spontaneous bleeding episodes [5].

Hemophilia is traditionally regarded as a familial genetic disorder transmitted through female carriers. As a deleterious mutation that reduces individual fitness, such variants, especially those with severe HA, would be expected to diminish over time through natural selection, particularly in the absence of adequate medical care [6]. However, the incidence of hemophilia A—estimated at 24.6 cases per 100,000 males for all severities and 9.5 cases for severe HA—has remained stable [5]. This persistence is largely attributable to sporadic mutations. Sporadic hemophilia, defined as the first affected individual in a family with no prior history of hemophilia or carrier status among relatives, accounts for approximately 30–60% of HA cases and represents a key mechanism for disease maintenance in the population [7,8].

Mutations in the *F8* gene are broadly categorized into inversion variants—primarily intron 22 and intron 1 inversions—and non-inversion variants (NIVs), which include point mutations, deletions, insertions, and duplications [9,10,11]. Inversion mutations have been extensively studied in sporadic hemophilia, with evidence indicating that they originate predominantly but not exclusively in male germ cells [12,13,14,15]. Our recent investigations into NIVs revealed that HA sporadic NIVs are primarily caused by de novo mutations. With clear definitions of “occurrence”—the proband or the carrier mother, and haplotypic “origin”—the family member who transmitted mutant *F8* variant to the offspring (occurrence) but does not himself/herself harbor the variant and exhibits no coagulation abnormalities, HA sporadic NIVs arise through two distinct events—grandparental (Event 1) and maternal (Event 2). Furthermore, based on our data, the origin of sporadic NIVs is most often from female, contrary to inversion variant. In addition, our clinical observation suggested that the sporadic NIV seemed to be an isolated event; no recurrence in same generation within the family was reported [16,17].

This study is the continuation of previous studies aiming to thoroughly address how frequently de novo mutations recur in the same generation within the same family. Based on sibship inheritance analysis from all available family pedigrees registered in multiple medical centers in Taiwan, we concluded that HA sporadic NIVs exhibit very low recurrence risk.

## 2. Results: Sibship Inheritance Analysis Indicates Sporadic HA Variants Rarely Recur in the Same Generation

We analyzed 24 comprehensive family pedigrees from the 126 hemophilia A families registered in multiple medical centers in Taiwan. (Table 1). A pedigree was deemed adequate if it included grandparents, parents, and proband. Linkage analysis was performed using four intragenic markers (F8int9.2, F81VS13, F8int21, F8IVS22) and an extragenic marker (DSX9901) to determine X-chromosome inheritance patterns.

Of the 24 pedigrees studied, 13 families contained at least one individual from the same generation as the sporadic HA occurrence (either the proband or the carrier mother), thereby enabling inheritance analysis. In two families (Table 1, Families 1 and 4), a member inherited the homologous, non-mutant X chromosome rather than the X chromosome on which the HA-NIV occurred. In the remaining 11 families, 16 individuals inherited chromosomes identical to those bearing the HA-causing *F8* mutation in the sporadic HA occurrence.

**Figure 1:**
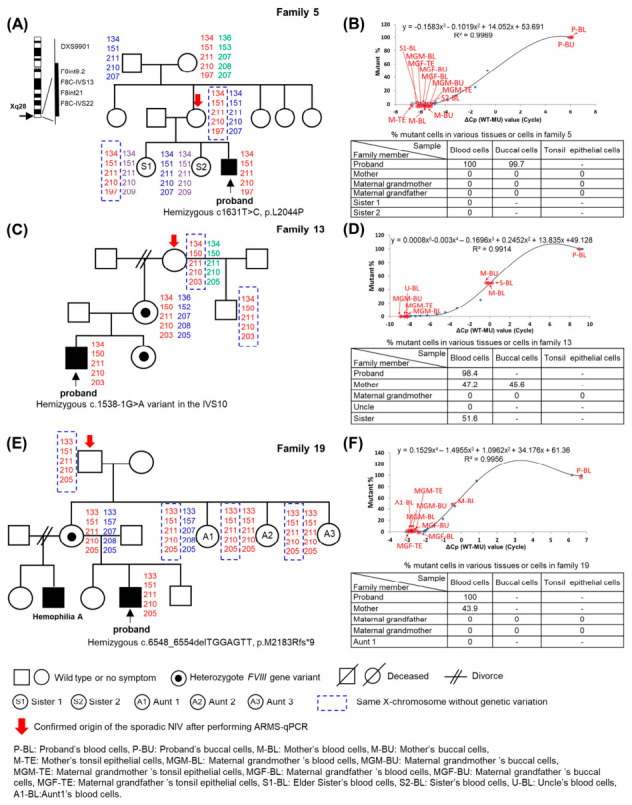
Sibship inheritance analysis by linkage analysis and ARMS-PCR. (**A**,**C**,**E**) Linkage analysis using intra- and extragenic markers for the *F8* gene to determine the X chromosome inheritance patterns of the sporadic non-inversion variants (NIV) in family 5 (severe hemophilia A), family 13 (moderate hemophilia A), and family 19 (severe hemophilia). The *F8* gene located on the X chromosome (Xq28) is shown on the left side of Figure 1A, along with the four intragenic markers and one extragenic marker (F8int9.2, F8IVS13, F8int21, F8IVS22, and DXS9901) selected for linkage analysis. The same X chromosome from family members as the X chromosome bearing hemophilia A-causing mutant in the occurrence was framed. (**B**,**D**,**F**) ARMS-qPCR was applied in various tissue cells obtained from family members to determine the percentage of mutant cells and was shown in each table.

Amplification refractory mutation system-quantitative polymerase chain reaction (ARM-qPCR), a customized PCR method that differentially amplifies mutant and wild-type alleles with enhanced sensitivity for detecting low levels of mutant cells (<0.1%), was used to detect the presence of *F8* variants in the 16 analyzable individuals. Among the 16 individuals, none carried the associated *F8* variant (Table 1, Figure 1). For example,

- Family 5 (Figure 1A): The mother (origin) transmitted the same X chromosome to the elder sister of the proband (occurrence). Despite inheriting the same chromosome, the chromosome exhibited wildtype *F8* allele without the associated hemophilia-causing genetic change in the proband. The proband’s other sister inherited the homologous X chromosome from the mother.
- Family 13 (Figure 1B): The maternal grandmother (origin) transmitted the hemophilia-causing chromosome to proband’s mother (occurrence, carrier). As for the proband’s uncle, although he inherited the same X chromosome from the maternal grandmother, his X chromosome exhibited wildtype *F8* allele.
- Family 19 (Figure 1C): The maternal grandfather (origin) transmitted the same X chromosome to the proband’s mother (occurrence, carrier) and her three sisters. Despite inheriting the same chromosome, all three sisters exhibited wildtype *F8* alleles.

**Figure 2:**
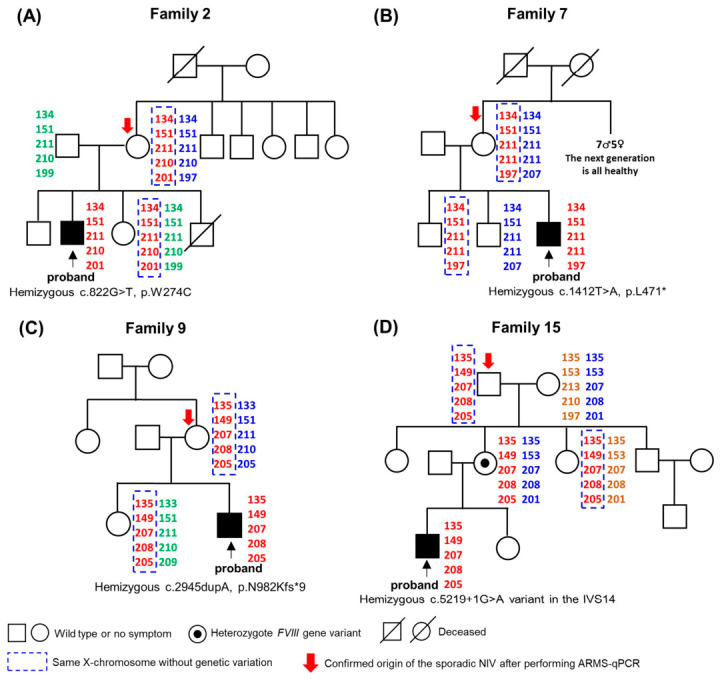
Linkage analysis of Family 2, 7, 9 and 15 (**A**–**D**) using intra- and extragenic markers for the *F8* gene to determine the X chromosome inheritance patterns. The same X chromosome from family members as the X chromosome bearing hemophilia A-causing mutant in the occurrence was framed.

Results of linkage analysis for Family 2, Family 7, family 9 and Family 15 were shown in Figure 2. No sibling recurrence was observed.

Results of linkage analysis and ARMS-qPCR for Family 3, Family 12, Family 16 and Family 21 have been previously reported [16].

In summary, under the applied detection methods, no sibling recurrence was observed, no additional carriers sharing the same X haplotype were identified, and no somatic mosaicism was detected in tested tissues. These findings from a total of 11 families strongly suggest that the recurrence risk of sporadic *F8* gene non-inversion variants in the same generation is very low.

## 3. Discussion

Sporadic hemophilia A enables the persistence of HA in the population. HA is generally considered a genetic disease propagated through female carriers. Unlike HA with clear family history where recurrence risk can be clearly estimated, determining the origin, occurrence and estimating recurrence risk are major challenges in genetic counseling for sporadic hemophilia [18].

A mutation-specific gender ratio exists for inversional mutation [12,13]. Over 90% of *F8* inversions, which are presented in 40–50% of HA sporadic variants, originate from males [13,19]. For *F8* non-inversional variants, based on our study [17] and previous studies [20,21], no such male dominance could be observed.

The de novo mutation, either inversion variant or non-inversion variant, can arise during gametogenesis (sperm/egg formation), early embryonic, or postzygotic development [16,18].

When a de novo mutation originates from a grandparent (Event 1), the affected female is typically an obligate carrier, though mosaicism may occasionally occur [22]. Rarely, mosaicism has been reported in asymptomatic males [23,24,25,26], see also Supplementary Materials Table S1 and Figure S1).

In Event 2, where the mutation originates from the proband’s mother, it likely occurs during post-zygotic, first few cell divisions, rendering the mother unlikely to be a carrier. Among 11 analyzable families, the X chromosome bearing the sporadic HA non-inversion variant (NIV) was transmitted to 11 occurrences (probands, heterozygote carrier mothers, and mosaic carrier mothers). However, the same X chromosome was inherited by 16 additional offsprings from the same generation without the associated genetic variation. Based on the Poisson model for rare events (rule of three), when zero recurrence events are observed among N families, the upper 95% confidence bound for recurrence risk can be approximated as 3/N. The approximated current risk in the current study (3/11) is significantly lower than the current risk in familiar HA with female carriers (50%).

Nevertheless, it is important to acknowledge the limitation of the current study that isolated germline mosaicism cannot be definitively excluded in family members identified as the origin of sporadic mutations, and isolated germline mosaicism can pass down mutations to an offspring and be misinterpreted as de novo mutations [27]. Patients with mosaicism should be considered as an “occurrence” in the current study instead of an “origin”. Among the six families whose origins of sporadic HA were confirmed to be the maternal grandfather, one sperm sample was available (family 16), and ARMs-qPCR confirmed 0% of mutant cells [16], supporting a true de novo mutation. As maternal germ cells cannot be directly studied, isolated germline mosaicism cannot be definitely ruled out in females assigned as the origin of sporadic mutation. However, by reviewing our data from hemophilia registries, we have identified four families with germline mosaicism for HA mutations (families 21–22 [16], and family 25 [26], see also Supplementary Materials Table S1 and Figure S1); all of them exhibited somato-germline mosaicism. Although isolated germline mosaicism has been reported in other genetic disorders, including von Willebrand disease [28], and suggested in HA by Kasper [8] and Lannoy [29], these findings in HA might require confirmation using more sensitive methodologies to rule out somato-germline mosaicism.

Accurate determination of maternal carrier status is essential when only one affected son has been born. A true carrier mother—either heterozygous or mosaic—faces a genuine and measurable risk of recurrence, whereas a sporadic case, based on our data, rarely recur. From a genetic counseling point of view, the overall recurrence risk is positively correlated with the level of somatic mosaicism in parental blood [27,30], and negative results from somatic cell testing have not excluded occult germline mosaicism. As zero recurrence risk cannot be guaranteed, current guidelines recommend avoiding definitive statements regarding carrier status and suggest discussing prenatal diagnosis in subsequent pregnancies [18,31]. In the era of highly sensitive prenatal diagnostic technologies and advanced therapies that enable individuals with HA to lead near-normal lives, a more positive and encouraging approach to childbearing, combined with diligent prenatal screening, may represent an appropriate strategy.

## 4. Materials and Methods

### 4.1. Patients and Family Groups and Study Design

This study was conducted under the Declaration of Helsinki and approved by the Institutional Review Board of Changhua Christian Hospital (approval no. 201209). Only patients who bore HA sporadic NIVs were investigated. All patients and relevant family members provided informed consent. Sporadic patients were recruited from 2015 to 2024 from among 126 registered families with HA to prevent selection bias. Only families containing three generations of data (patients to MGPs) were analyzed.

The occurrence of sporadic HA is defined as the first affected patient with hemophilia or a hemophilia carrier in a family where no relative or cousin has hemophilia or has been proven to be a hemophilia carrier. The haplotypic origin of sporadic HA is defined as the family member who transmitted the mutant *F8* variant to the offspring (the occurrence) but does not himself/herself harbor the variant and exhibits no coagulation abnormalities. The possible origin of sporadic HA NIV was identified by genetic testing and linkage analysis (4.2) and confirmed by amplification refractory mutation system-quantitative polymerase chain reaction (ARMS-qPCR, 4.3). Genetic testing, linkage analysis and ARMS-qPCR were also applied to evaluate sibship inheritance patterns in the current study.

### 4.2. Linkage Analysis for Sibship Inheritance Analysis

Intragenic (F8int9.2, F8IVS13, F8int21 and F8IVS22) and closely located extragenic (DXS9901) HA gene markers (short tandem repeat elements) were used to trace the X chromosomal inheritance patterns as previously described [16,32,33].

### 4.3. Amplification Refractory Mutation System-Quantitative Polymerase Chain Reaction (ARMS-qPCR)

A specialized form of PCR that enables differential amplification of mutant and wildtype alleles with heightened sensitivity for detecting mutant (<0.1%), was performed as previously described [16,17].

## 5. Conclusions

Based on long-time clinical observation and thorough sibship inheritance analysis from all available family pedigrees registered in multiple medical centers in Taiwan, among 11 families and 16 relevant family members, sporadic non-inversion variants exhibit very low recurrent risk in the same generation within families. Our findings provide insightful information for genetic counseling in sporadic non-inversional hemophilia A.

## Figures and Tables

**Table 1:** Characteristics of HA-NIVs and sibship inheritance analysis.

Family No.	FVIII Level (IU/dL)	Amino Acid Substitution	Family Members Designated as the Origin of Sporadic NIV ^§^	Number of Family Members from the Same Generation as the Occurrence of Sporadic NIV (Proband or Carrier Mother) Bearing the Same X-Chromosome as the Occurrence	Number of Family Members from the Same Generation as the Occurrence of Sporadic NIV (Proband or Carrier Mother) Bearing *F8* Variant
1	<1	p.R2016G	M	0	0 ^¶^
2	<1	p.W274C	M	1	0
3	<1	p.R550C	M	1	0
4	3.7	p.R1708C	M	0	0 ^¶^
5	<1	p.L2044P	M	1	0
6	1	p.N1460Ifs*5	M	0	0 ^¢^
7	<1	p.L471*	M	1	0
8	<1	p.D135N	M	0	0 ^¥^
9	<1	p.N982Kfs*9	M	1	0
23	<1	p.I1213Ffs*5	M	0	0 ^¥^
24	7.8	p.R2169H	M	0	0 ^¥^
10	<1	p.N982Kfs*9	MGM	0	0 ^¥^
11	<1	p.Y1781*	MGM	0	0 ^¥^
12	<1	p.I1213Ffs*5	MGM	2	0
13	1.2	c.1538-1G>A in the IVS10	MGM	1	0
14	<1	p.P617Sfs*7	MGF	0	0 ^¥^
15	<1	c.5219+1G>A in the IVS14	MGF	1	0
16	<1	p.Y605H	MGF ^†^	1	0
17	<1	p.Q774Hfs*12	MGF	0	0 ^¥^
18	<1	p.I1213Nfs*28	MGF	0	0 ^¢^
19	<1	p.M2183Rfs*9	MGF	3	0
20	<1	p.R509*	MGM ^‡^	0	0 ^¢^
21	25.1	p.R546W	MGF ^‡^	3	0
22	<1	p.S62*	EGT M ^‡^	0	0 ^¥^

M: mother; MGM: maternal grandmother; MGF: maternal grandfather; EGT, earlier generation than; § tissue cells (blood cells, buccal cells, and tonsil epithelial cells) obtained from each of them all demonstrated 0% mutant cells by amplification refractory mutation system-qualitative polymerase chain reaction; † sperm cells also exhibited 0% mutant cells; ‡ their descendants (mother) demonstrated mosaic variants. ¶ bearing homologous X-chromosome. ¢ no family members available for testing. ¥ family member unwilling to be tested.

## Data Availability

The data reported in this study are available upon request from the corresponding author. This data is not publicly available due to ethical restrictions.

## Supplementary Materials

The following supporting information can be downloaded at: https://www.mdpi.com/article/10.3390/ijms27093831/s1.
