# Supplementary material for: Non-Inversion Variants in Sporadic Hemophilia A Rarely Recur
Source: Int J Mol Sci. 2026 Apr 25;27(9):3831. doi: 10.3390/ijms27093831 (PMC13164367; doi:10.3390/ijms27093831)
Supplement: Supplementary file 1 [file ijms-27-03831-s001.zip › ijms-4174325-supplementary.pdf]

TableS1. Characteristics of hemophilia A sporadic mosaic noninversion variants.

Family No. and Age of Proband (Year)	Family File No.	FVIII Level (IU/dL)	Exon	Nucleotide Change	Amino acid Substitution	Family Members Designated as the Possible Origin of Sporadic NIVs	Percentage of Mutant Cells for Family Members Designated as the Possible Origin of Sporadic NIVs			Family Members Designated as the Confirmed Origin of Sporadic NIVs	Percentage of Mutant Cells for Family Members Designated as Confirmed Origin of Sporadic NIVs		
							Blood Cells	Buccal Cells	Tonsil Epithelial Cells		Blood Cells	Buccal Cells	Tonsil Epithelial Cells
20 (10)	153	<1	10	c.1525A>T	p.R509*	M	18.1	23.8	24.1	MGM	0	0	0
21 (38)	130	25.1	11	c.1636C>T	p.R546W	M	7.3	5.1	2.8	MGF	0	0	0
22 (47)	105	<1	2	c.185 C>G	p.S62*	M	9.4	3.7	5.4	EGT M	NA	NA	NA
25 (5.5)	149	<1	14	c.3637delA	p.I1213Ffs*5	MGF	24.5	19.4	2.7	EGT M	NA	NA	NA

M, mother; MGM, maternal grandmother; MGF, maternal grandfather; EGT, earlier generation than; NA: not available.

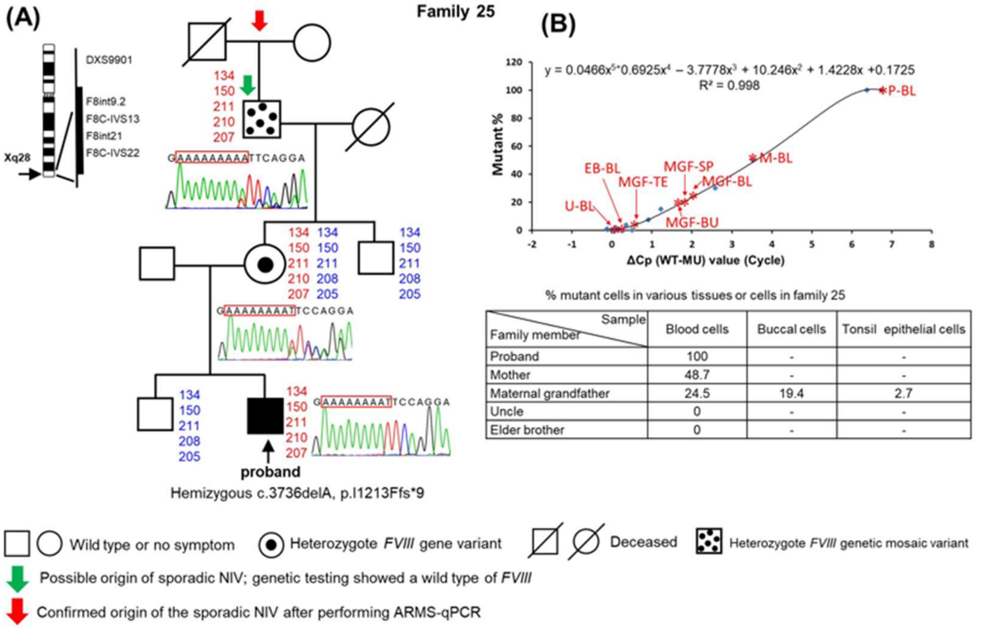

Sample / Family member	Blood cells	Buccal cells	Tonsil epithelial cells
Proband	100	-	-
Mother	48.7	-	-
Maternal grandfather	24.5	19.4	2.7
Uncle	0	-	-
Elder brother	0	-	-

P-BL: Proband's blood cells, M-BL: Mother's blood cells, MGF-BL: Maternal grandfather 's blood cells, MGF-BU: Maternal grandfather 's buccal cells, MGF-TE: Maternal grandfather 's tonsil epithelial cells, MGF-SP: Maternal grandfather 's sperm cells, U-BL: Uncle's blood cells, EB-BL: Elder brother's blood cells.

Supplementary **Figure S1**: Germline and somatic mosaicisms of factor VIII gene mutation identified in an asymptomatic grandfather. (A)A sporadic severe hemophilia A (FVIII: C <0.1 IU/dl) was diagnosed in a 5.5-year-old boy. Genetic analysis revealed a hemizygous mutation c.3637delA, pI1213F fs*5 in the exon 14. Genetic analysis revealed the carrier status of proband's mother (48.7%). Genetic analysis and ARMS-qPCR (B)revealed that proband's maternal grandfather had a mosaic mutant that occurred in 24.5%, 19.4% and 2.7% in blood cells, buccal cells, and tonsil epithelial cells respectively.
